# Group 1 Metabotropic Glutamate Receptor Function and Its Regulation of Learning and Memory in the Aging Brain

**DOI:** 10.3389/fphar.2012.00182

**Published:** 2012-10-12

**Authors:** Caroline Ménard, Rémi Quirion

**Affiliations:** ^1^Department of Psychiatry, Douglas Mental Health University Institute, McGill UniversityMontreal, QC, Canada

**Keywords:** metabotropic glutamate receptors, aging, learning, memory, synaptic plasticity

## Abstract

Normal aging is generally characterized by a slow decline of cognitive abilities albeit with marked individual differences. Several animal models have been studied to explore the molecular and cellular mechanisms underlying this phenomenon. The excitatory neurotransmitter glutamate and its receptors have been closely linked to spatial learning and hippocampus-dependent memory processes. For decades, ionotropic glutamate receptors have been known to play a critical role in synaptic plasticity, a form of adaptation regulating memory formation. Over the past 10 years, several groups have shown the importance of group 1 metabotropic glutamate receptor (mGluR) in successful cognitive aging. These G-protein-coupled receptors are enriched in the hippocampal formation and interact physically with other proteins in the membrane including glutamate ionotropic receptors. Synaptic plasticity is crucial to maintain cognitive abilities and long-term depression (LTD) induced by group 1 mGluR activation, which has been linked to memory in the aging brain. The translation and synthesis of proteins by mGluR-LTD modulate ionotropic receptor trafficking and expression of immediate early genes related to cognition. Fragile X syndrome, a genetic form of autism characterized by memory deficits, has been associated to mGluR receptor malfunction and aberrant activation of its downstream signaling pathways. Dysfunction of mGluR could also be involved in neurodegenerative disorders like Alzheimer’s disease (AD). Indeed, beta-amyloid, the main component of insoluble senile plaques and one of the hallmarks of AD, occludes mGluR-dependent LTD leading to diminished functional synapses. This review highlights recent findings regarding mGluR signaling, related synaptic plasticity, and their potential involvement in normal aging and neurological disorders.

## Aging and Cognition

Over the next decades, a majority of developing countries will face one of the biggest challenges in history: accelerated aging of its populations. In human, aging is characterized by physical, psychological, and social changes. Interestingly, major individual differences are known to exist and low probability of disease and disability, high cognitive and physical capacity, and active engagement in life in general are associated to successful aging. Several animal models have been developed to investigate the processes behind this phenomenon (Gallagher et al., 2011). Indeed, it is important to understand not only how to increase lifespan but maintain quality of life.

One of the most dreaded and studied feature of aging is related to cognitive dysfunctions. Therefore, behavioral studies of rodent animal models are useful to explore mechanisms underlying memory deficits associated to aging. The Morris Water Maze (MWM) test was introduced in 1984, to study hippocampus-dependent memory in rats (Morris, 1984). The hippocampus is crucial for working memory and particularly spatial learning. Briefly, a platform is hidden under opaque water and the animal has to learn the platform position using visual clues over a daily training period. Aging generally affects the time the rat will take to reach the platform, when performances are compared to young animals. For the Long-Evans rat strain however, a sub-group of old animals maintains high cognitive abilities despite aging while another sub-group shows mild cognitive impairments, thus making this model suitable to explore successful aging processes (Gallagher et al., 1993; Menard and Quirion, 2012). Cognition is not limited to spatial memory. Reactivity to novelty and gustatory/olfactory memory, another form of cognition involving multiple neuronal networks and brain areas, is also deficient in old memory-impaired Long-Evans rats, while aged memory-unimpaired animals cope well with environmental changes (Rowe et al., 1998). Other forms of memories have not yet been tested in this rat strain. While cognition include more than formation of memories, we chose to focus on learning and consolidation of long-term memories for this review. For more information on other cognitive processes in aged rat models such as attention, rule reversal, discrimination, working memory, and the different circuits involved, please refer to Gallagher et al. (2011). Multiple rat models should be studied to unravel the impact of aging on cognition. Indeed, Sprague-Dawley rats show faster acquisition than Long-Evans rats for a five-choice serial reaction time examining multiple aspects of cognition and executive functions (Auclair et al., 2009). Aging could differentially affect these processes.

Early on, alterations in cholinergic neuronal activity were linked to memory impairments in the Long-Evans rat model (Aubert et al., 1995). More recently, gene expression has been compared as a function of age and cognitive status since changes in transcription are related to age-associated spatial learning impairments (Burger et al., 2007; Haberman et al., 2011). Several targets have been highlighted including immediate early genes, as well as transthyretin, quinone reductase 2, and prodynorphin (Rowe et al., 2007; Benoit et al., 2011). In addition, dysregulation of epigenetic controls during aging (Penner et al., 2010) could alter the expression of genes involved in memory formation.

An interesting hypothesis to explain age-associated cognitive deficits is the inability to encode new information because of aberrant neuronal networks (Wilson et al., 2006). Neurogenesis has been reported in the adult hippocampus and could be involved in consolidation of new memories. The number of adult progenitor and neural stem cells decline with age (Villeda et al., 2011). Moreover, abnormal differentiation of newborn granule cells have been linked to age-related memory impairments in Agouti rats (Nyffeler et al., 2010). Exercise prevents cognitive deficits by increasing neurogenesis and decreasing apoptosis in the old hippocampus (Kim et al., 2010a). Surprisingly, greater hippocampal neurogenesis has been correlated to memory deficits in old Long-Evans rats (Bizon and Gallagher, 2005). This intriguing observation could either suggest impaired differentiation processes or underlying high cellular stress. Indeed, inflammatory pathways become hyperactivated with age and dysfunctions of the immune system promote neurodegeneration (Lucin and Wyss-Coray, 2009; von Bernhardi et al., 2010). Higher neurogenesis could counteract this phenomenon in the old memory-impaired Long-Evans rat hippocampus. To support this hypothesis, preserved neuron number, total number of synapses, and synaptic proteins have been previously reported in the hippocampus of this animal model (Rapp and Gallagher, 1996; Nicolle et al., 1999, Geinisman et al., 2004). On the other hand, an alteration in post-synaptic densities (PSD) protein expression (Nyffeler et al., 2007) and a reduction in PSD area have been associated to memory impairments in aged Long-Evans rats (Nicholson et al., 2004). Refined cellular and molecular mechanisms could lead to a successful cognitive aging and facilitate the maintenance of memory capacities despite aging.

## The Biochemistry Behind Memory: Glutamate Receptors and Synaptic Plasticity

**Figure 1:**
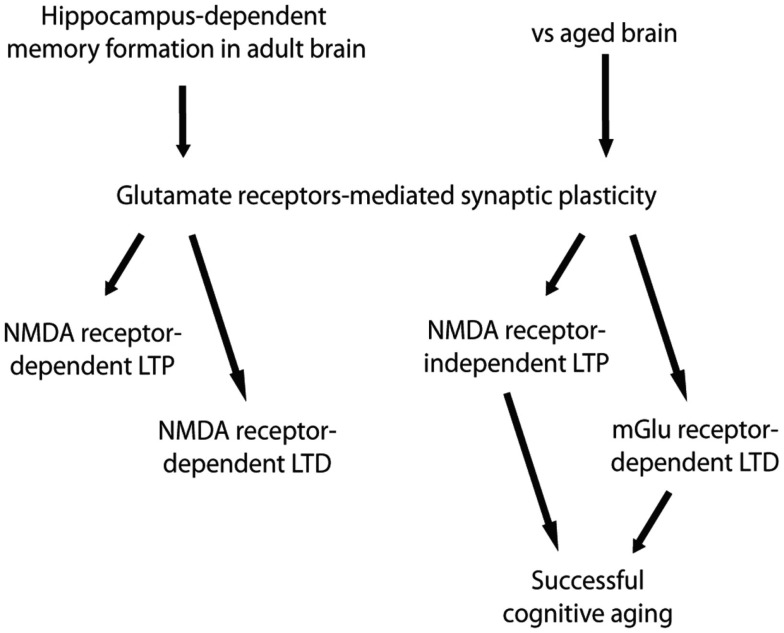
**Memory formation and synaptic plasticity in adult versus aged rat brain**. Young and adult rats strengthen or weakened their neuronal networks interconnections using various adaptative mechanisms including NMDA receptor-dependent long-term potentiation (LTP) and long-term depression (LTD). On the other hand, successful cognitive aging has been associated with NMDA-independent LTP and mGluR-dependent LTD in memory-unimpaired Long-Evans aged rats.

The formation of new memory traces involves complex networks of neurons in various brain areas. The hippocampus plays a central role in working memory and short-term cognition while cortices store and organize souvenirs following consolidation (Frankland and Bontempi, 2005). While spatial memory is highly dependent on the hippocampus, novel object recognition, and reference memory, cognition processes also affected by aging, involve perirhinal cortex (Burke et al., 2010). Acquisition of a reference memory could be independent of the hippocampus, for example, acquisition of fear conditioning may solely depend on the amygdala, although it may be rendered hippocampal-dependent through manipulation of experimental parameters. The adaptative mechanisms involved require to strengthen or weaken interconnections of neuronal networks known as synaptic plasticity. The biochemistry of synaptic plasticity related to learning and memory has been extensively studied over the last few decades (Baudry et al., 2011). The excitatory neurotransmitter glutamate and its receptors are closely involved in those processes especially for hippocampus-dependent spatial memory (Lee and Silva, 2009). Glutamate receptors are present on the membrane of both neurons and glial cells and can be classified into two distinct groups: ionotropic and metabotropic. The ionotropic glutamate receptors family are ion channels activated by glutamatergic agonists and include the 2-amino-3-hydroxy-5-methyl-isoxaole-4-propinate (AMPA) and *N*-methyl-d-aspartate (NMDA) receptor subtypes (Baudry et al., 2011). The stimulation of AMPA and NMDA receptors induces synaptic plasticity leading to a long-term potentiation (LTP), a form of long-lasting enhancement in signal transmission (Lee and Silva, 2009; Baudry et al., 2011). Those receptors allow Ca^2+^ ions to enter into the cell, activating several intracellular signaling pathways, receptor trafficking, gene expression, and LTP production (Lee and Silva, 2009; Baudry et al., 2011). Changes in Ca^2+^ homeostasis have been associated with age-related memory impairments (Burke and Barnes, 2010). In fact, the induction and maintenance of LTP is compromised during aging as reported by several groups (Barnes, 2003; Kumar, 2011). In the Long-Evans rat model, cognitive performances of aged memory-unimpaired animals have been strongly correlated with the magnitude of NMDA-independent LTP (Boric et al., 2008). While young rats seem to rely on NMDA-LTP to build new memories, aged rats use different mechanisms (Figure 1). One reason behind this intriguing phenomenon could be the decrease in ionotropic receptor subunits generally occurring with age (Zhao et al., 2009). The Lou/C/Jall rat strain, however, is an exception to this rule; characterized by an incredible lifespan, absence of obesity, and of various severe pathologies related to aging (Alliot et al., 2002), these animals maintain high cognitive abilities, ionotropic glutamate receptor levels, excitatory postsynaptic currents (EPSC) and LTP despite aging (Kollen et al., 2010).

Long-term depression (LTD), another form of synaptic plasticity has been closely related to memory formation (Ge et al., 2010). LTD has not been investigated as much as LTP in this context but mechanisms behind LTD-associated synaptic plasticity are starting to be unraveled. Contrary to LTP, LTD reduces the efficacy of neuronal synapses in several areas of the brain including the hippocampus (Collingridge et al., 2010). LTD could be important to develop effective neuronal network connections, to facilitate the generation of spatial cognitive maps, and to erase old memory traces (Kemp and Manahan-Vaughan, 2007; Malleret et al., 2010). Prolonged stimulation of NMDA receptors reduces the density of receptors in the PSD, leading to LTD (Mulkey and Malenka, 1992; Collingridge et al., 2010). The activation of G-protein-coupled metabotropic glutamate receptors (mGluR) can also induce LTD (Palmer et al., 1997; Bellone et al., 2008). Interestingly, NMDA receptor-independent LTD has been correlated with successful cognitive aging in Long-Evans rats (Lee et al., 2005).

## Group 1 Metabotropic Glutamate Receptors and Successful Aging

Cloning of the first mGluR subtype was realized in the early 1990s (Masu et al., 1991). Eight mGluR have since been identified and divided into three groups: group 1 with mGluR1 and mGluR5, group 2 includes mGluR2 and mGluR3, and finally group 3 with mGluR4, mGluR6, mGluR7, and mGluR8 (Nicoletti et al., 2011). LTD can be induced by applying group 1 specific agonists such as 3,5-dihydroxyphenylglycine (DHPG; Palmer et al., 1997). In the context of this review, we will focus on this group even if antagonism of pre-synaptic group 2 mGluRs can modulate LTD and spatial memory (Altinbilek and Manahan-Vaughan, 2009).

mGlu1 receptors have been associated with the post-synaptic specialization of excitatory synapses and are concentrated in perisynaptic and extrasynaptic areas (Nicoletti et al., 2011). On the other hand, mGluR5 functionally interact with NMDA receptors in the PSD (Tu et al., 1999; Collett and Collingridge, 2004). mGlu5 receptors are abundant in the hippocampus and cerebral cortex in the adult brain (Romano et al., 1996). Mice lacking mGluR5 show impaired learning and reduced LTP in the CA1 region (Lu et al., 1997). The EPSC of AMPA receptors are normal in these mice while the LTP component related to NMDA receptors is completely lost (Jia et al., 1998). This deficit can be rescued by protein kinase C (PKC) stimulation (Jia et al., 1998). mGluR5 potential role in learning and memory was confirmed by gene targeting (Jia et al., 2001). mGluR5 knock-out (KO) mice show mild cognitive deficits in the MWM task whereas memory impairments were significant for discrimination reversal, which involves inhibitory learning processes necessary to extinct old memories (Xu et al., 2009). Accordingly, we reported an increase in mGluR5 protein levels in the PSD of aged memory-unimpaired Long-Evans rats and inhibitory learning was highly conserved in this group in comparison with old memory-impaired animals (Menard and Quirion, 2012). These behavioral and biochemical data are in agreement with higher mGluR-LTD previously reported in aged memory-unimpaired Long-Evans rats (Lee et al., 2005). Aging is associated with pattern separation deficits: old Fischer 344 rats show age-dependent object recognition memory impairment (Burke et al., 2010). Novel object recognition involves perirhinal cortex but we speculate that the confusion between old versus new platform positions reported for aged memory-impaired Long-Evans rats in the hippocampus-dependent MWM task could be explained by alterations in synaptic processes modulated by mGluR. Indeed, the stimulation of group 1 mGluR could act like a molecular switch to facilitate synaptic plasticity (Bortolotto et al., 2005) and to regulate late phases of both LTP and LTD in the hippocampus (Neyman and Manahan-Vaughan, 2008). Daily application of the mGluR5 antagonist, 2-methyl-6-(phenylethynyl) pyridine (MPEP), impaired both working and reference memory in Wistar rats while only working memory is affected in hooded Lister rats (Manahan-Vaughan and Braunewell, 2005). This difference could be related to higher brain levels of mGluR5 in Wistar rats (Manahan-Vaughan and Braunewell, 2005). Prolonged pharmacological blockade of mGluR5 by MPEP reduces mGluR1 expression, impairs LTP as well as spatial memory, and suppresses theta and gamma oscillations in the dentate gyrus (Bikbaev et al., 2008). Increased neurogenesis in aged memory-impaired Long-Evans rat as mentioned previously could be related to disrupted neuronal networks modulated by mGlu5 receptors. Fear memory, gustatory memory, and social interaction could also be modulated by mGluR5 (Simonyi et al., 2010). Interestingly, addiction is often characterized by persistent cognitive deficits and Long-Evans rats treated with methamphetamine show reference memory impairments (Reichel et al., 2011). This deficit can be reversed by treatment with an allosteric modulator of mGlu5 receptors (Reichel et al., 2011). Memory processes are highly influenced by stress and various psychosocial factors (Kremen et al., 2012) and could influence group 1 mGluR interactions with its effectors and scaffolding Homer proteins (Tronson et al., 2010).

## Modulation of Group 1 Metabotropic Glutamate Receptor Function

The Homer proteins family consists of dendritic auxiliary proteins that contain a single PDZ domain and bind to the carboxy (C)-terminal domain of group 1 mGluR (Brakeman et al., 1997), acting as both scaffolding and transduction molecules (Fagni et al., 2002). Homer proteins are widely expressed in the central nervous system as well as in peripheral tissues and are predominantly localized at the PSD of mammalian neurons (Shiraishi-Yamaguchi and Furuichi, 2007). Consequence of alternative splicing, several variants of Homer products exist and are classified as either short (Homer 1a) or long (Homer 1b/c, Homer 2, and Homer 3) forms (Fagni et al., 2002). Long isoforms of Homer are constitutively expressed (Xiao et al., 1998) and form synaptic clusters with other proteins of the PSD, facilitating signal transduction, and interactions with plasma membrane receptors (Shiraishi-Yamaguchi and Furuichi, 2007). NMDA receptors for example are directly associated to mGluR via the interaction of Homer with Shank and PSD-95 protein complexes (Tu et al., 1999). Homer 1 proteins also regulate mGluR trafficking in the cell and therefore downstream signaling resulting from neuronal activity (Ango et al., 2002; Duncan et al., 2005). While Homer 1c increases cell surface expression of mGluR1 in the plasma membrane (Ciruela et al., 2000), Homer 1b retains mGluR5 in the endoplasmic reticulum (Roche et al., 1999). On the other hand, Homer 1a is an immediate early gene expressed following neuronal activity (Tu et al., 1998). When bound to group 1 mGluR, Homer 1a disrupts the protein clusters by competitive binding (Xiao et al., 1998; Shiraishi-Yamaguchi and Furuichi, 2007). Homer 1a acts as a dominant negative modulator by reducing mGluR5 coupling with its signaling effectors (Kammermeier and Worley, 2007). It may also inhibit NMDA currents following disruption of the Homer–Shank complex (Bertaso et al., 2010). Conversely, a prolonged activation of NMDA receptors increases Ca^2+^ entry in neuronal cells, activating multiple enzymes including proteases such as calpains (Zadran et al., 2010). Calpains cleave mGluR1 C-terminal domain and maintain their ability to release cytosolic Ca^2+^ from intracellular stores resulting in excitotoxicity and eventually neuronal degeneration (Xu et al., 2007). Interestingly, the ratio of Homer 1a to Homer 1b can modulate mGluR-mediated plasticity of AMPA receptor transmission in optic cells following visual stimulation (van Keuren-Jensen and Cline, 2006). mGluR receptors and Homer proteins could also be involved in synaptogenesis (Xiao et al., 2000) and actively modulate synaptic plasticity in learning and memory. Thus, an overexpression of Homer 1a in the dorsal hippocampus may impair spatial working memory (Klugmann et al., 2005; Celikel et al., 2007).

Another intriguing hypothesis is about the involvement of mGlu receptors in homeostatic scaling (Hu et al., 2010). This form of non-Hebbian plasticity reinforces synaptic strength or activity of neuronal networks; the strength of synapses either scaled up by diminishing the surrounding network activity or scaled down by increasing this same network (Turrigiano and Nelson, 2000). An increment or reduction of AMPA receptors at excitatory synapses is an effective common form of homeostatic scaling (O’Brien et al., 1998; Turrigiano and Nelson, 2000). The activation of group 1 mGluR and the synthesis of Homer 1a regulate the expression of AMPA receptors. The level of Homer 1a is up-regulated after neuronal activity followed by agonist-independent signaling of group 1 mGluR, which reduces the tyrosine phosphorylation of GluR2 AMPA receptor subunit, subsequently scaling down the expression of those plasma membrane receptors (Hu et al., 2010).

Norbin, a key neuronal regulator promoting neurite outgrowth, synaptic plasticity, and LTP, also interacts with mGluR5 (Wang et al., 2010). This neuron-specific protein not only physically interacts with mGluR5 but increases the cell surface localization of the receptor and subsequently mGluR5 signaling (Wang et al., 2009). Similar to mGluR5 KO mice, Norbin KO animals show impaired synaptic plasticity and altered behaviors (Wang et al., 2009).

Group 1 mGluR regulate the activity of several kinases. However, their direct phosphorylation affects both receptor signaling and trafficking (Kim et al., 2008; Mao et al., 2008). The stimulation of both mGluR1 and mGluR5 triggers the release of Ca^2+^ intracellular stores. While mGluR1 activation by glutamate induces a single peak of Ca^2+^ release, mGluR5 releases Ca^2+^ by oscillations (Kawabata et al., 1996). This mechanism is made possible by the phosphorylation by PKC at residue threonine 840 of mGluR5 (Kawabata et al., 1996). On the other hand, the phosphorylation of serine 901 by PKC inhibits mGluR5 binding to calmodulin, decreasing its surface expression (Lee et al., 2008). Moreover, PKC can phosphorylate mGluR5 at multiple sites, quickly desensitizing the receptor (Gereau and Heinemann, 1998). Cyclin-dependent kinase 5, a proline-directed serine/threonine kinase can also phosphorylate the Homer binding domain of both mGluR1 and mGluR5, enhancing the interaction (Orlando et al., 2009).

## Signaling Pathways Related to Group 1 mGluR Activation

Ionotropic and mGluRs can act synergistically to induce LTP and to modulate synaptic plasticity. For example, the activation of both receptors seems to be required for the stimulation of the PKCgamma (γ) isoform (Codazzi et al., 2006). Spatial experience and training increases PKCγ expression (Nithianantharajah and Murphy, 2009), which is subsequently translocated from the cytoplasm to the membrane and is co-localized with mGluR5 in the CA1 hippocampus region after receptor activation (Liu et al., 2008). Individual differences in cognitive status observed in aged Long-Evans rats have been linked to this kinase (Colombo et al., 1997; Colombo and Gallagher, 2002). Moreover, the activation of PKC in small groups of hippocampal or cortical neurons in 2-year-old rats improved MWM performance, confirming the important role of this kinase in learning and spatial memory (Zhang et al., 2009). PKC enzyme activators have been proposed to enhance cognitive performances and may prevent or reduce dementia and memory deficits associated to age-related neurodegenerative diseases (Sun and Alkon, 2010).

Protein kinase C is not the only group of kinases regulated by ionotropic and mGluRs function; NMDA and mGluR5 receptors also interact to regulate the phosphorylation of the extracellular signal-regulated protein kinase (ERK; Yang et al., 2004). This synergy is related to PSD-95 and Homer 1b/c interaction and is independent of Ca^2+^ influx. The activation of ERK leads to the facilitation of c-fos expression, an immediate early gene produced following neuronal activity (Yang et al., 2004). Accordingly, ERK signaling is required to establish mGluR-dependent LTD in the hippocampus (Gallagher et al., 2004) and it diminished with age (Williams et al., 2006). The inhibition of the ERK signaling pathway does not impair MWM learning acquisition but prevent long-term spatial memory formation (Blum et al., 1999). Thus, age-related changes in ERK expression could be involved in cognitive decline and lower mGluR function may exacerbate this deficit.

**Figure 2:**
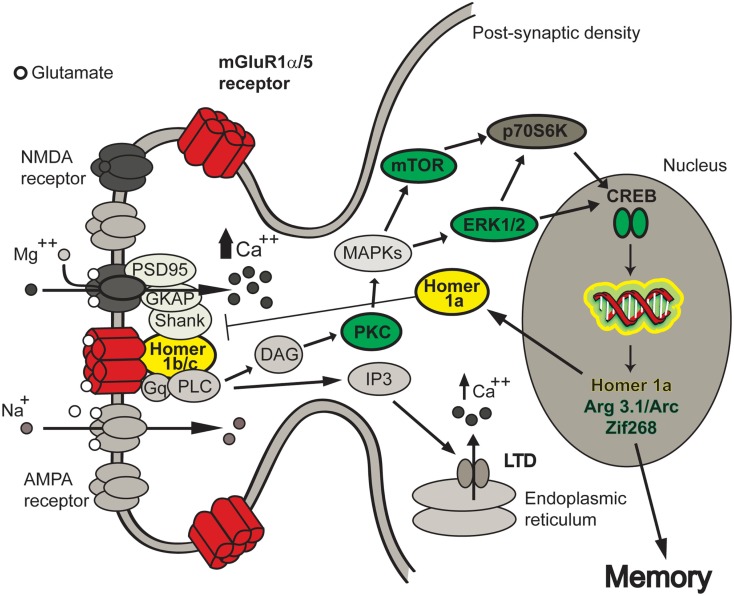
**Signaling pathways involved in mGluR-LTD and hippocampus-dependent spatial memory**. Group 1 metabotropic glutamate receptors (mGluR1α/5) are bound to ionotropic glutamate receptors by multiple scaffolding proteins (Homer 1 b/c, Shank, GKAP, PSD-95). Following mGluR stimulation, several protein kinases including PKC, ERK1/2, mTOR, and CREB become activated and induce the expression of various immediate early genes like Homer 1a, Arg3.1/Arc, and Zif268. Homer 1a is released in the cytosol and translocated to the plasma membrane uncoupling the mGlu receptors from its signaling effectors. These signaling pathways have been associated with multiples brain functions and could be critical to maintain cognition during normal aging.

The stimulation of group 1 mGluR with DHPG increases that of another central kinase for multiple signaling pathways: the mammalian target of rapamycin (mTOR; Page et al., 2006). The inhibition of mTOR prevents mGluR-LTD induced by DHPG (Hou and Klann, 2004) and our group has recently shown a strong positive correlation between successful cognitive aging in Long-Evans rats and downstream signaling pathways of group 1 mGluR activation including the mTOR cascade (Menard and Quirion, 2012). This serine/threonine protein kinase modulates the activity of several translation regulatory factors including p70S6 kinase (Page et al., 2006). An increased phosphorylation of the S6 complex has been associated to mGluR-LTD (Antion et al., 2008). Moreover, the activation of NMDA and mGluRs has been shown to regulate dendritic synthesis via mTOR activity (Gong et al., 2006). Hence, mTOR-dependent translational control is critical not only for spatial memory. Stability of long-term fear memory is compromised if mTOR activation and phosphorylation of p70S6 kinase is altered (Parsons et al., 2006). In addition, mTOR could contribute to alcohol abuse and addiction by up-regulating AMPA GluR1 subtype and Homer proteins in the nucleus accumbens (Neasta et al., 2010). Dysfunctions of downstream cell signaling events linked to mGlu receptors could thus impair multiple aspects of the brain functions (Figure 2).

Gene expression has often been targeted to explain memory deficits related to aging (Alberini, 2009; Benoit et al., 2011). Dysregulation of the phosphorylation of the cAMP response element-binding (CREB), a cellular transcription factor modulating the transcription of many genes, has been reported in the hippocampus of aged rats after fear conditioning training (Monti et al., 2005). CREB protein level is also lower in old memory-impaired rats compared to young animals (Brightwell et al., 2004). CREB-mediated transcription could stabilize the establishment and reactivation of spatial memory (Kim et al., 2011). Reduced synthesis of several proteins related to LTP and among proteins regulated by CREB, the activity-regulated cytoskeleton-associated protein (Arc, also known as Arg 3.1) has been correlated to aged-related cognitive impairments (Monti et al., 2005; Menard and Quirion, 2012). Conversely, calcineurin, a phosphatase modulating CREB activation, is highly expressed in the hippocampus of aged rats (Monti et al., 2005) and negatively correlated to LTP and memory (Malleret et al., 2001). Recently, our group reported that lower hippocampal CREB phosphorylation levels in old memory-impaired Long-Evans rats following MWM training could lead to reduced Arc expression and cognitive deficits (Menard and Quirion, 2012).

Following neuronal activity, Arc encoding mRNA is quickly transported to dendrites and then translated, making it a strong candidate for synaptic plasticity underlying LTP and LTD induction and maintenance (Bramham et al., 2008; Shepherd and Bear, 2011). Homeostatic scaling has recently been associated to Arc expression (Beique et al., 2011) and our group proposed that successful cognitive aging is linked to high levels of Arc expression in hippocampal dendrites of aged rats following mGluR stimulation by training (Menard and Quirion, 2012). Interestingly, activity-induced Notch signaling pathway in neurons also requires Arc expression (Alberi et al., 2011). Notch plays an important role in cell fate specification and could contribute to memory formation. Indeed, the activation of Notch signaling pathway prevents neural stem cells depletion and maintains tight control of neurogenesis in the adult brain (Imayoshi et al., 2010). Accordingly, reduced Arc and Notch signaling could be closely related to memory impairments associated with aging.

Another immediate early gene, Zif268, is required for the late phases of LTP and the expression of long-term memories. Indeed, LTP is lost after 24 h in mutant mice (Jones et al., 2001; Bozon et al., 2002). Zif268 is constitutively expressed in the rat brain with high levels seen in the hippocampal CA1 region (Alberini, 2009). While the role of Arc in synaptic plasticity seems to cover several phases of memory formation, the expression of Zif268 is apparently critical for specific steps like the persistence of LTP and the reactivation of old memories (Bozon et al., 2003). In our animal model of successful cognitive aging, we reported no significant changes in Zif268 levels (Menard and Quirion, 2012). While the role of Zif268 in associative memory is still debated, conflicting results have also been reported regarding synaptic plasticity (Alberini, 2009).

Group 1 mGluR signaling could be crucial for cognition during aging but it has also been related to pathological conditions like Fragile X syndrome (FXS) and Alzheimer’s disease (AD). Results published on animal models of those diseases and clinical trials conducted with patients will thus help to understand the processes involved in the aging of the brain.

## mGluR Receptor Malfunction and Fragile X Syndrome

**Figure 3:**
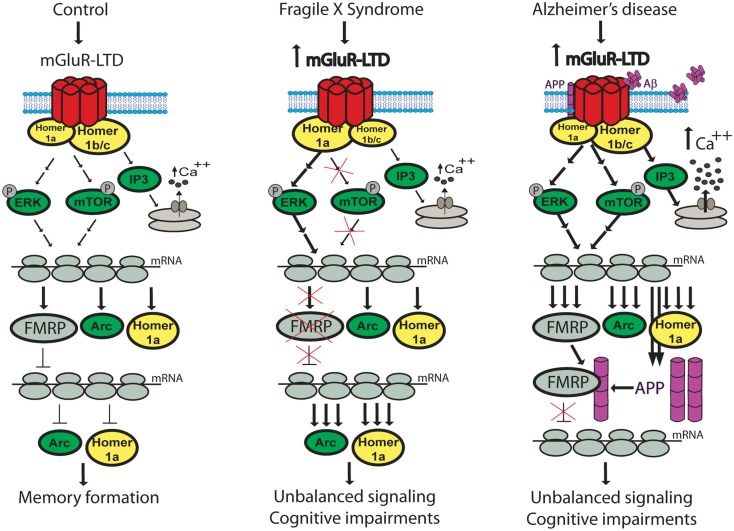
**Comparison of mGluR-related signaling pathways activation in successful cognitive aging, Fragile X syndrome, and Alzheimer’s disease**. Activation of group 1 mGluR mediates Ca^+2^ release from intracellular stores and protein translation through the activation of several kinases including ERK and mTOR. Once translated, FMRP proteins act as a negative regulator of protein synthesis and reduce LTD-related proteins levels and IEG expression. A good balance of these mechanisms leads to memory formation in the aged brain. Excessive mGluR-LTD has been linked to the Fragile X syndrome. While ERK activation is enhanced, protein synthesis related to the mTOR pathway is lost. Moreover, mGluR5 is associated more with Homer 1a stimulating agonist-independent signaling pathways. Without FMRP to decrease the protein translation, LTD-related proteins are overexpressed and memory is impaired. Excessive mGluR-LTD has also been reported in Alzheimer’s disease. Aβ oligomers clusters mGluRs on the plasma membrane promoting Ca^2+^ signaling and release from intracellular stores. Stimulation of group 1 mGluR also increases APP translation. FMRP can bind to APP possibly altering the protein synthesis negative control and creating memory deficits.

Group 1 mGluRs have been implicated in the development of the FXS (Dolen et al., 2007). FXS is the most common inherited cause of mental retardation and is likely related to autism (Dolen and Bear, 2008). A mutation blocks the *FMR1* gene transcription which encodes the Fragile X mental retardation protein (FMRP; Pieretti et al., 1991). This RNA-binding protein acts predominantly as a negative regulator of protein synthesis and controls the dynamic translation of several mRNA involved in learning and memory including Arc (Park et al., 2008; Darnell et al., 2011). FMRP regulates several mRNA localization, stability, and translational efficiency in dendrites following mGluR stimulation (Zukin et al., 2009). Multiple symptoms are associated with FXS including moderate to severe mental retardation, behavioral problems, dysmorphic features, and increased susceptibility to seizures (Dolen and Bear, 2008). NMDA-dependent LTP is reduced in the anterior piriform cortex of adult and aged FXS mice (Larson et al., 2005; Gocel and Larson, 2012). On the other hand, synaptic plasticity related to LTP and NMDA-dependent LTD is intact in FMRP KO mice hippocampus (Godfraind et al., 1996). Accordingly, protein levels of AMPA and NMDA glutamate receptor subtypes are unaltered as well (Giuffrida et al., 2005). In contrast, mGluR5 level is specifically decreased in the PSD, suggesting reduced protein synthesis induced by the stimulation of group 1 mGluR (Giuffrida et al., 2005). This alteration could explain the cognitive impairments observed for inhibitory learning and reverse memory in the MWM task (Godfraind et al., 1996). Unfortunately, this last finding has never been replicated by other groups. Conversely, mGluR-LTD is enhanced in these FMRP mutant mice (Huber et al., 2002). Whereas mGluRs seem to play an important role in successful cognitive aging (Menard and Quirion, 2012), young animals favor NMDA-dependent synaptic plasticity processes (Lee et al., 2005). Hence, excessive mGluR-LTD in adult *FMR1* KO mice and FXS patients could dysregulate synaptic plasticity and lead to memory deficits (Luscher and Huber, 2010; Figure 3).

The stimulation of group 1 mGluR with the specific agonist DHPG normally induces FMR protein translation in dendrites (Weiler et al., 1997). In *FMR1* KO mice, the lack of this protein synthesis suppressor may unbalance cellular mechanisms and exaggerate mGluR-dependent signaling (Dolen and Bear, 2008). Hence, excessive protein synthesis downstream of mGluR activation could induce the complex synaptic pathophysiology seen in FXS (Bear et al., 2004). In fact, mRNA translation seems to be hypersensitive to basal ERK activation in the absence of FMRP and contributes to seizure susceptibility (Osterweil et al., 2010). Phosphoinositide 3-kinase (PI3K), an enzyme downstream of several cell surface receptors including group 1 mGluR, becomes excessively active and insensitive to mGluR stimulation in the absence of FMRP (Gross et al., 2010). In contrast, mGluR-simulated protein translation initiated by the mTOR pathway is lost (Ronesi and Huber, 2008). These alterations are probably linked, at least in part, to disrupted Homer scaffolds. Indeed, in the *FMR1* KO mice brain, mGluR5 is associated more with Homer 1a stimulating agonist-independent signaling pathways than long Homer isoforms (Ronesi et al., 2012). Genetic deletion of Homer 1a rescues mGluR signaling, enhances protein translation and reduces the severity and incidence of seizures in this FXS animal model (Ronesi et al., 2012). On the other hand, Homer 1a deletion did not improve abnormal mGluR-LTD or DHPG-induced Arc synthesis deficit, supporting an essential role of FMR protein in these processes (Ronesi et al., 2012). In accordance, in *FMR1* KO mice, mGluR-LTD formation does not require the acute stimulation of protein synthesis which is probably related to the constitutive overexpression of proteins implicated in LTD formation and maintenance to compensate for the lack of *FMR1* (Hou et al., 2006; Nosyreva and Huber, 2006). Homeostatic plasticity is affected as well in this FXS animal model with a decrease in the expression of AMPA surface receptors (Nosyreva and Huber, 2006). The magnitude of LTP can be enhanced following group 1 mGluR stimulation; this facilitation is known as LTP priming (Cohen and Abraham, 1996). In *FMR1* KO mice, LTP priming is reduced by mGluR decoupling from protein synthesis (Auerbach and Bear, 2010).

The first demonstration of mGluR modulation rescuing phenotypes in FXS animal models was attained by pharmacologically rescuing social interaction and memory impairments in the *Drosophila* fly model (McBride et al., 2005). Age-dependent cognitive deficits can also be rescued by mGluR antagonists in this animal model (Choi et al., 2010). Decreased mGluR5 signaling could rescue several features in the FXS mutant mice as well including dendritic spine abnormalities, ocular dominance plasticity, accelerated body growth, open field center behavior, and seizure susceptibility (Yan et al., 2005; Dolen et al., 2007). Synaptic plasticity deficits including enhanced mGluR-LTD can be reversed by chronic *in vivo* treatment with group 2 mGluR antagonists in these mice (Choi et al., 2011). Profound cognitive impairments are observed in human suffering from FXS. Interestingly, alterations in inhibitory avoidance extinction can be reversed in *FMR1* KO mice by reducing mGluR-LTD with a 50% reduction in mGluR5 expression (Dolen et al., 2007). Since reducing group 1 mGluR function improves multiple phenotypes in animal models of FXS, clinical trials based on mGluR5 inhibition are currently underway (Krueger and Bear, 2011) and the first results seem promising (Jacquemont et al., 2011). However, considering the potential role of these receptors in successful cognitive aging, potential side effects on memory processes should be carefully monitored.

## Alzheimer’s Disease and Group 1 mGluR Function

A possible link between FXS and AD has been proposed since FMR protein binds to the amyloid precursor protein (APP; Sokol et al., 2011), APP is overexpressed in the *FMR1* KO mice brain (D’Agata et al., 2002) and the group 1 mGluR agonist DHPG increases APP translation (Westmark and Malter, 2007; Sokol et al., 2011). Moreover, a strong genetic interaction has been identified between *Drosophila* FMRP gene and presenilin, a protein mutated in the familial form of AD (McBride et al., 2010). Reduction of presenilin function in this animal model induces age-related cognitive deficits that can be prevented by mGluR antagonists or genetic reduction (McBride et al., 2010). AD is the most common form of dementia and generally diagnosed in patients over 65 years of age. First described in 1906, this neurodegenerative disorder is characterized by mood changes, language dysfunctions, progressive cognitive deficits, and brain atrophy (Gauthier and Poirier, 2008; Pasic et al., 2011). The exact causes of AD are not well understood but include various hallmarks such as senile plaques and neurofibrillary tangles. These markers are used at the autopsy to confirm the diagnosis (Gauthier and Poirier, 2008; Pasic et al., 2011). Senile plaques consist of extracellular deposits of amyloid-beta (Aβ) proteins often surrounded by active microglia (Kellner et al., 2009). Aβ plaques tend to aggregate and become neurotoxic for the neighboring cells. In contrast, neurofibrillary tangles are made of hyperphosphorylated tau proteins and accumulate intracellularly leading to synaptic dysfunction (Gauthier and Poirier, 2008; Pasic et al., 2011). Since the stimulation of group 1 mGluR releases Ca^2+^ from intracellular stores, it can also facilitate the hyperphosphorylation of tau by up-regulating protein kinase activities (Tsai et al., 2005; Figure 3). Interestingly, the stimulation of group 1 mGluR with DHPG induces a rapid accumulation of APP c-terminal fragments (CTF) in synaptoneurosomes obtained from the brains of the CRND8 Alzheimer mouse model whereas group 2 mGluR activation triggers the production and release of Aβ (Kim et al., 2010b). In neuronal cultures, the selective activation of mGlu2 receptors amplify Aβ toxicity while the co-activation of the same receptors with mGluR3 is neuroprotective (Caraci et al., 2011). Significant mGlu2 receptor losses have been reported to occur in the enthorinal cortex in AD brains (Richards et al., 2010). The discrete localization of group 1 mGluR has not yet been investigated in detail in the human brain but its signaling is down-regulated in the frontal cortex of AD subjects and worsens with the progression of the disease (Albasanz et al., 2005). In rat hippocampal neurons, Aβ oligomers cluster at the cell surface and overstabilize mGluR5 in a time dependent manner, promoting increased Ca^2+^ signaling, synaptotoxicity, and eventually synapse deterioration (Renner et al., 2010). Concomitantly, soluble Aβ disrupts Homer 1b and Shank scaffolding with metabotropic and ionotropic glutamate receptors, respectively, shrinking the PSD and affecting downstream signaling pathways (Roselli et al., 2009). The activation of mGluR5 increases the translocation of receptors from extrasynaptic sites to the PSD, then Homer stabilizes the receptors in clusters in the plasma membrane (Serge et al., 2002). Both receptors and mGluR-Homer clusters are highly dynamic in their movements and this mobility is affected by Aβ leading to unbalanced mGluR-mediated signaling, protein translation, synaptic plasticity and eventually memory deficits. Thus, misfolded proteins in AD may not only induce pathology-related synaptic defects but also block the maintenance of successful cognitive aging processes. Recently, an enhanced endocannabinoid signaling has been reported in the hippocampi of patients suffering from AD, particularly around senile plaques (Mulder et al., 2011). Robust hippocampal LTD could be induced by activation of the pre-synaptic cannabinoid-1 receptors and this form of synaptic plasticity, downstream of NMDA, and mGlu5 receptor activation (Izumi and Zorumski, 2012), could be exacerbated by Aβ. Thus, it seems that various neurotransmission systems might be dependent of an intact mGluR function during aging.

## Conclusion

After several decades of intense studies on processes involving ionotropic glutamate receptors in the aging brain, their metabotropic counterparts, and particularly post-synaptic group 1 mGluRs are becoming key targets to unravel synaptic plasticity processes underlying learning and memory. mGlu receptors, Homer proteins, and downstream signaling pathways likely all play critical roles in the maintenance of high cognitive abilities in old age. Further studies are thus highly warranted especially considering their apparent role in FXS and possibly AD.

## Conflict of Interest Statement

The authors declare that the research was conducted in the absence of any commercial or financial relationships that could be construed as a potential conflict of interest.
